# The role of metabolic tumor volume (MTV) measured by [18F] FDG PET/CT in predicting EGFR gene mutation status in non-small cell lung cancer

**DOI:** 10.18632/oncotarget.16806

**Published:** 2017-04-04

**Authors:** Ao Liu, Anqin Han, Hui Zhu, Li Ma, Yong Huang, Minghuan Li, Feng Jin, Qiuan Yang, Jinming Yu

**Affiliations:** ^1^ School of Medicine, Shandong University, Jinan, China; ^2^ Department of Radiation Oncology, Shandong Cancer Hospital Affiliated to Shandong University, Jinan, China; ^3^ Department of Nuclear Medicine, Shandong Cancer Hospital Affiliated to Shandong University, Jinan, China; ^4^ Department of Radiation Oncology, Qilu Hospital Affiliated to Shandong University, Jinan, China

**Keywords:** non-small cell lung cancer, [18F] FDG PET/CT, epidermal growth factor receptor, mutation

## Abstract

Many noninvasive methods have been explored to determine the mutation status of the epidermal growth factor receptor (EGFR) gene, which is important for individualized treatment of non-small cell lung cancer (NSCLC). We evaluated whether metabolic tumor volume (MTV), a parameter measured by [18F] fluorodeoxyglucose positron emission tomography/computed tomography (PET/CT) might help predict EGFR mutation status in NSCLC. Overall, 87 patients who underwent EGFR genotyping and pretreatment PET/CT between January 2013 and September 2016 were reviewed. Clinicopathologic characteristics and metabolic parameters including MTV were evaluated. Univariate and multivariate analyses were used to assess the independent variables that predict mutation status to create prediction models. Forty-one patients (41/87) were identified as having EGFR mutations. The multivariate analysis showed that patients with lower MTV (MTV≤11.0 cm3, p=0.001) who were non-smokers (p=0.037) and had a peripheral tumor location (p=0.033) were more likely to have EGFR mutations. Prediction models using these criteria for EGFR mutation yielded a high AUC (0.805, 95% CI 0.712–0.899), which suggests that the analysis had good discrimination. In conclusion, NSCLC patients with EGFR mutations showed significantly lower MTV than patients with wild-type EGFR. Prediction models based on MTV and clinicopathologic characteristics could provide more information for the identification of EGFR mutations.

## INTRODUCTION

Treatment options for NSCLC, the most predominant type of lung cancer, have developed rapidly due to the discovery and investigation of genetic drivers such as EGFR-activating mutations [1, 2]. Mutations in EGFR act as both biomarkers and rational targets for treatment [2]. Patients with adenocarcinoma histology, females, never-smokers, and those of Asian ethnicity are more likely to have EGFR mutations and thus exhibit better responses to EGFR tyrosine kinase inhibitors (TKIs) [3]. Exon 19 deletions and mutations of exon 21 (L858R) are the two most prevalent (approximately 90%) activating mutations, and patients with these mutations have shown a high overall response rate to TKIs (approximately 80%) [2, 4, 5].

As a result, EGFR genotype could help in the selection of patients who will benefit from TKIs when making treatment decisions. However, some hurdles must still be overcome before making this individualized approach to treatment a reality. First, there is no unified mutation detection approach for testing patients. Other problems, such as tumor site inaccessibility, a shortage of tissues for testing, tumor heterogeneity or a patient's refusal to undergo invasive detection, also pose limitations. Thus, the development of noninvasive and effective methods to help identify the status of the EGFR gene is necessary. We previously discussed the role of PET/CT in staging, assessing therapy response and developing a radiotherapy plan for patients with NSCLC [6, 7]. PET/CT is based on the fact that the glucose metabolism of a tumor is partly reflected by FDG uptake. Processes downstream of the EGFR gene could influence glucose metabolism by regulating the synthesis of glucose-transporter-1 (GLUT1), which correlates with FDG uptake [8, 9]. Therefore, FDG uptake might be associated with EGFR gene mutation in tumors.

Many researchers have reported that the SUVmax of a primary lesion, a metabolic parameter on PET, is associated with prognosis in NSCLC [10–13]. Previous studies found that NSCLC patients who were treated with TKIs and who have a low SUVmax of the primary lesion might have better outcomes [14]. Given that patients with EGFR mutation showed a better response to TKIs than those with wild-type EGFR, it was hypothesized that low SUVmax might be associated with EGFR gene mutations [15]. Many researchers have focused on the relationship between PET/CT-associated parameters and EGFR gene mutation [15–25]. However, most prior studies focusing on SUVmax have produced varying results.

MTV is acquired by outlining a primary tumor using an SUVmax cutoff of 2.5, which provides additional information such as tumor burden and heterogeneity and could be prognostic for survival and tumor metabolic activity [20, 26–28]. We therefore explored the role of MTV for predicting EGFR mutation status in NSCLC and further established a useful prediction model to help in screening and identification of mutation status.

## RESULTS

### Patients and tumor characteristics

We identified 87 (mean age, 60 years) patients who underwent EGFR mutation detection. Adenocarcinoma was the major pathology (n=78; 89.7%). Male sex (n=49; 56.3 %), non-smoker status (n=55; 63.2 %), advanced stage (III–IV) (n=74; 85.1 %) and peripheral tumor location (n=59; 67.8 %) were relatively predominant. Forty-one of the patients (47.1 %) had EGFR mutations, and the main mutation subtypes were exon19 deletion (n=14) and exon 21 L858R point mutation (n=25). Other mutations were identified in the two other patients with an EGFR mutation. Clinical factors and EGFR mutation status are summarized in Table 1 and Table 2.

**Table 1:** Patient Characteristics and tumor variables

	Number (%)
Patients, n	87 (100)
Age (years)	
Median	60
Range	29-86
Gender, n	
Male	49 (56)
Female	38 (44)
Smoking status, n	
Smoker	32 (37)
Never-smoker	55 (63)
Stage, TNM, n	
I/II	13 (15)
III/IV	74 (85)
Pathology, n	
ADC	78 (90)
Other	9 (10)
Location, n	
Peripheral	59 (68)
Central	28 (32)

**Table 2:** Clinical characteristics and EGFR mutation status

Variables	Total	EGFR+ (%)	EGFR- (%)	P
Age				
>60	41	19(46)	22(53)	1.000
≤60	46	22(48)	24(52)	
Gender				
Male	49	22(45)	27(55)	0.670
Female	38	19(50)	19(50)	
Smoking status, n				
Smoker	32	8(25)	24(75)	0.002
Never-smoker	55	33(60)	22(40)	
Stage, AJCC, n				
I/II	13	8(62)	22(48)	0.368
III/IV	74	33(45)	41(55)	
Pathology, n				
ADC	78	40(51)	38(49)	0.032
Other	9	1(11)	8(89)	
Location, n				
Peripheral	59	34(58)	25(42)	0.006
Central	28	7(25)	21(75)	
Diameter, n				
>3.5 cm	36	13(36)	23(64)	0.126
≤3.5 cm	51	28(55)	23(45)	
SUVmax, n				
>10.4	46	20(43)	26(57)	0.470
≤10.4	41	21(51)	20(49)	
SUVmean, n				
>6.0	43	10(23)	34(77)	0.087
≤6.0	44	31(72)	12(28)	
MTV, n				
>11.0 cm^3^	44	10(23)	34(77)	0.001
≤11.0 cm^3^	43	31(72)	12(28)	
CEA				
>15.0 ng/mL	42	22(52)	20(48)	0.382
≤15.0 ng/mL	42	17(49)	25(60)	

Abbreviations: SUVmax: maximal standardized uptake value; MTV: metabolic tumor volume; EGFR+: EGFR mutation; and EGFR-: no EGFR mutation.

### Comparison of metabolic parameters and EGFR mutation status

**Figure 1:**
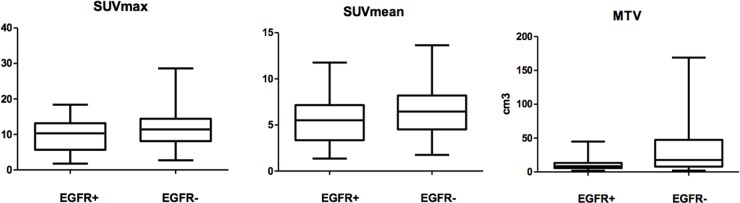
Comparison of metabolic parameters of primary lesions in NSCLC between EGFR+ and EGFR- by Wilcoxon rank-sum test SUVmean and MTV, p<0.05; SUVmax p >0.05.

Patients with EGFR mutations had significantly lower MTV and SUVmean values than those with wild-type EGFR (p=0.001 and p=0.031, respectively) (Figure 1). No statistically significant correlation with SUVmax was found between patients with mutant and wild-type EGFR.

### EGFR status and clinical features

A significant correlation was found between EGFR status and smoking history, pathology and tumor location. Other characteristics, including age, gender, and TNM stage, did not differ significantly by mutation status. ROC curve analysis revealed a pretreatment CEA cutoff and tumor diameter of 8.0 (ng/mL) and 3.5 (cm), respectively, with AUCs of 0.57 and 0.65. However, no significant difference was found according to these two factors (Table 2).

### EGFR status and PET/CT results

The comparison of different EGFR mutation statuses and metabolic parameters is shown in Figure 1. There was no association between the tested metabolic parameters and EGFR mutation status. We therefore explored the use of metabolic parameters in predicting EGFR mutation status. ROC curve analysis revealed cutoff points for SUVmax, SUVmean and MTV of 10.4, 6.0 and 11.0 (cm^3^), respectively, with AUCs of 0.599, 0.634 and 0.711. We dichotomized the patients according to these thresholds and found that EGFR mutations were more frequent in patients with a lower MTV (p=0.001), indicating the predictive role of this parameter.

### Predicting EGFR mutation

**Figure 2:**
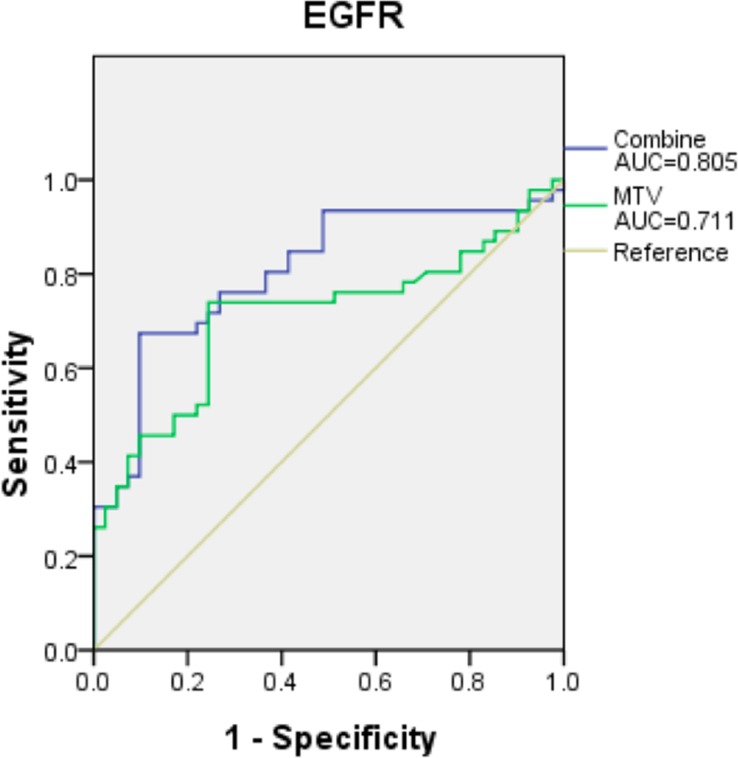
The prediction models consist of three criteria MTV, non-smokers and peripheral location for EGFR mutation yielded a higher AUC (0.805, 95% CI 0.712-0.899, *p*=0.001), which suggests that the model has good discrimination. However, the AUC when using only MTV to predict EGFR mutation was lower.

In univariate analysis, EGFR mutation was correlated with ADC pathology, non-smoker status, peripheral tumor location, and low MTV. In addition, tumor diameter was an important factor in our study because EGFR mutations tended to be relatively more frequent in patients with smaller-diameter tumors, although no statistically significant difference (p=0.126) was found between the two groups. Multivariable logistic regression analysis with inclusion of these parameters revealed that only non-smoker status, peripheral tumor location and low MTV were significant predictors of EGFR mutation (Table 3). ROC curve analysis was then performed to validate the predictive value of these factors; an AUC of 0.805 was produced, which suggests good discrimination (Figure 2). The sensitivity and specificity of our prediction model including MTV were 61.0% and 80.4%, respectively.

**Table 3:** Multivariate regression analyses for various predictive factors of EGFR mutation

	EGFR		
	OR	95% CI	P
Never smoker	3.589	1.077-11.953	0.037
ADC	2.288	0.211-24.822	0.496
Peripheral location	3.833	1.113-13.207	0.033
MTV≤11.0 cm^3^	35.859	4.038-318.481	0.001
Diameter≤3.5 cm	0.134	0.015-1.209	0.073

## DISCUSSION

Individual gene detection has been recommended for advanced NSCLC; however, such assessment is often limited by tumor inaccessibility, insufficient sample tissue for detection and patients’ unwillingness to undergo invasive detection procedures [15]. Therefore, PET/CT is advantageous as a noninvasive strategy for predicting EGFR gene mutation status. In this study, we found that MTV, a metabolic parameter estimated by PET/CT, was correlated with EGFR mutation status. By contrast, SUVmax did not show a significant correlation.

Many prior studies have focused on the role of PET/CT in predicting EGFR mutation status. Na et al. and Huang et al. found a significant association between SUVmax and EGFR mutation status, although the trends between the EGFR mutant groups and wild-type groups in the two reports differed [15, 16]. Three other studies found no relationship between SUVmax and EGFR gene mutation status [19, 20, 24]. Data from previous association studies are summarized in Table 4.

**Table 4:** Summary of published data on the associations between EGFR mutation and variables in patients with NSCLC

Author	Year	Country	Number of Patients	TNM stage	Pathology	Mutations	FDG-Variables	Findings	Other variables
Na	2010	Korea	100	1, 2, 3, and 4	ADC+SCC +Other	19 and 21	SUVmax	Low SUVmax was predictive of EGFR mutations.	N
Huang	2010	China	77	3 and 4	ADC	18, 19, 20, and 21	SUVmax	High SUVmax was predictive of EGFR mutations.	N
Mak	2011	America	100	1, 2, 3, and 4	ADC+SCC +Other	18, 19, 20, and 21	SUVmax	Low SUVmax was predictive of EGFR mutations.	Smoking history
Choi	2012	Korea	163	3 and 4	ADC+SCC +other	18, 19, 20, and 21	SUVmax, SUVmean	Low SUVmax was predictive of EGFR mutations	Smoking history
Putora	2013	Switzerland	14	NA	ADC	19 and 21	SUVmax	Not predictive of EGFR mutations.	N
Chung	2014	Korea	106	1, 2, 3, and 4	ADC	18, 19, 20, and 21	SUVmax and tMTV	Not predictive of EGFR mutations	NA
Caicedo	2014	Spain	102	3 and 4	ADC+SCC +other	18, 19, 20, and 21	SUVmax	Not predictive of EGFR mutations	N
Ko	2014	China	132	1, 2, 3, and 4	ADC	18, 19, 20, and 21	SUVmax	High SUVmax was predictive of EGFR mutations.	CEA, smoking history, and diameter
Lee	2015	China	71	4	ADC	18, 19, 20, and 21	nSUVmax and mSUVmax	nSUVmax, mSUVmax were predictive of EGFR mutations	Age, gender, and smoking history
Mo	2015	Korea	206	1, 2, 3, and 4	ADC+SCC +Other	18, 19, 20, and 21	SUVmax	Not predictive of EGFR mutations	Gender and smoking history
Cho	2016	Korea	61	1, 2, 3, and 4	ADC+SCC +other	18, 19, 20, and 21	SUVmax	SUVmax was predictive of EGFR mutations	Gender

Abbreviations: SUVmax: maximal standardized uptake value of primary lesion; nSUVmax: maximal standardized uptake value of metastasis lymph node; mSUVmax: maximal standardized uptake value of metastasis lesions (except lymph node); tMTV: metabolic tumor volume of all malignant lesions throughout the body; ADC: adenocarcinoma; and SCC: squamous cell carcinoma.

According to our observations, the presence of EGFR mutations did not correlate with SUVmax or SUVmean. A possible explanation for these observations is that SUVmax and SUVmean are semi-quantitative indexes that could vary with different PET scanners, fasting duration, level of plasma glucose and region of interest (ROI) parameters. In addition, simple SUV might fail to reflect the spatial features and behaviors of a primary lesion in imaging, which could provide more information about the biological behaviors of tumors such as mutation status and tumor heterogeneity [29].

Given the limitation of SUVmax, we chose another volume-based PET/CT parameter, metabolic tumor volume (MTV), as an alternative variable to explore the relationship between PET/CT and EGFR gene mutation status. This parameter provides complementary information about disease burden, which is prognostic for outcomes and tumor metabolic activity [27, 30]. Previous studies have found a positive impact of low MTV on patient outcomes [20, 26, 31]. It is also well known that patients with mutant EGFR appear to have a better prognosis than patients with wild-type EGFR [2, 4, 5]. Thus, we hypothesize that low MTV may be associated with EGFR gene mutation.

Some prior studies focused on this parameter have reported that whole MTV is not associated with EGFR gene mutation status [20, 32]. Whole MTV includes all malignant lesions and can be influenced by substantial interference. Focusing only on the MTV of a primary lesion, as in the current work, might reduce this interference to a certain extent. Using different criteria, such as SUV2.5, SUV3.0, and SUV40%, to define MTV could influence results, but SUV2.5 has been identified as the best choice [33]. We found that low MTV was associated with EGFR mutation and established that MTV is predictive of EGFR gene mutations.

Our sample size (n=87) was comparable to previous similar studies, which have ranged from 77 to 106 in size. Furthermore, clinicopathological variables, such as sex, tumor histology, smoking history, and tumor location, were explored in our study, and a peripheral location and smoking history were found to be associated with EGFR mutation. These factors have varied in former studies, which could be a result of evaluating different numbers of patients and patients from different countries. Our findings provide evidence that a low MTV is associated with EGFR mutation and that PET/CT-associated parameters have a role in the noninvasive prediction of EGFR gene mutation status.

Noninvasive examination has been a focus of many recent studies, including our own. Determining how to utilize a patient's clinicopathologic and imaging information to help identify EGFR mutation is worth studying. Measurements of metabolic parameters such as MTV and SUVmax offer an easy pathway towards this goal, as the majority of patients receive PET/CT during primary diagnosis and staging. Despite being readily available, however, the metabolic parameters that could help identify EGFR mutation have not been well researched to date. Based on the results in the current work, the sensitivity and specificity for our prediction model that included MTV were 61.0% and 80.4%, respectively.

One advantage of using metabolic parameters to predict EGFR mutation status is that these parameters provide direct measures of malignant lesions and can reflect tumor heterogeneity to a certain degree. However, many noninvasive methods can experience interference caused by the internal environment, and different technologies have different standards. By contrast, PET/CT is well established and has been widely used. Many studies have tried to identify useful parameters that could help predict EGFR mutation status using conventional imaging such as CT and MR. Our group has focused on the clinical application value of PET/CT in many fields. Our prediction model showed better discrimination than previous models and warrants further research. Finally, the results from the current work might be used to help develop an imaging biomarker to non-invasively identify EGFR mutation status using PET imaging to complement, but not replace, molecular testing. Prospective studies with blinded mutation status and independent datasets will be needed to further validate the predictive power of the metabolic parameters discussed here. Furthermore, additional studies should investigate how EGFR mutation gives rise to certain phenotypic traits that are quantified by these imaging parameters.

There were some limitations to our study. First, the study had a retrospective design with a relatively small size. Second, a possible bias could have existed in the process of patient-selection. Third, differences in metabolic parameters between EGFR mutation and mutations in other important genes (e.g., ALK) were not discussed, which is something we aim to address in future studies. We will continue to follow up with the patients assessed here and intend to publish survival results in the future. In addition, there were several PET/CT parameters that were not discussed. As heterogeneity in tumor phenotype can be quantitatively described through radiomic features, [29, 34, 35] we plan to assess these parameters in the future. Overall, the current work makes important contributions to the noninvasive prediction of EGFR mutation status in patients without a known genotype.

In conclusion, the results of the present study suggest that EGFR mutation in an Asian population with NSCLC is correlated with clinical and metabolic parameters, including MTV, smoking status and tumor location. The combined evaluation of these three factors could be helpful in discriminating mutation status, especially for patients with inadequate sampling or when genetic testing is not available. However, a larger, multi-institutional, prospective study is needed for further validation of the current results, and cost analysis is also mandatory when developing an optimal diagnostic algorithm.

## PATIENTS AND METHODS

### Study design and patients

The institutional review board approved this study for human investigation. Between January 2013 and September 2016, retrospective analysis was carried out for all newly diagnosed patients with pathologically confirmed NSCLC with further testing performed for EGFR mutation analysis and PET/CT examination less than 2 weeks before any treatment in Shandong Cancer Hospital Affiliated with Shandong University. Tumor samples of primary lesions were obtained by CT-guided biopsy, bronchoscopy, or pathologically or from postoperative specimens. We excluded the following patients: (1) patients who had any therapy prior to PET/CT, (2) patients whose specimens were inadequate for mutation analyses, (3) patients with acute and chronic pneumonia or other infections that might interfere with PET/CT imaging, (4) patients who had other cancers previously, (5) patients who had double or multiple primary cancers, and (6) patients whose primary lesion measured less than 1 cm in diameter, which might cause an error in PET/CT imaging due to a partial volume effect. Ultimately, clinical data and PET/CT imaging data were analyzed from 87 patients who underwent EGFR testing. Basic clinical characteristics are summarized in Table 1. Age, gender, smoking history, TNM stage, location of primary tumor (central or peripheral), maximum diameter of primary tumor according to CT, pre-therapy level of serum CEA (normal 0–3.4 ng/ml) and metabolic parameters from PET/CT were analyzed, as shown in Table 2. Patients who never smoked or smoked less than 100 cigarettes until the time of diagnosis were regarded as non-smokers. The others were considered smokers [23].

### PET/CT imaging analysis

Pretreatment PET/CT scans were performed using a PET/CT scanner (Discovery LS, GE Healthcare). The patients fasted for no less than 6 hours before the examination, and blood glucose levels met the requirement before intravenous injection of [18F] FDG. Sixty minutes later, PET and CT scans were obtained during free breathing with axial sampling at 4.25 millimeters thickness per slide. Reconstruction and analysis of PET and CT images were achieved using the manufacturer's review station (Xeleris; GE Healthcare).

Two experienced PET/CT physicians (M.L. and H.Y.) measured tumor SUVmax, SUVmean and MTV for all patients. MTV was defined as the volume of the part of the primary lesion that was obtained using the cutoff (SUV≥2.5), which has been widely approved for NSCLC [33]. SUVmax and SUVmean of the MTV were obtained automatically through the manufacturer's software. The details of the procedure have been reported previously [33].

### EGFR gene mutation test

Pathological samples used for mutation status analysis were obtained via surgery, bronchoscopy or CT-guided biopsy. Genomic DNA from tumor tissue was acquired from paraffin-embedded sections using a microdissection method based on the protocols recommended by the manufacturer. ARMS-PCR was used to amplify the EGFR gene, and detection was performed using an ADx EGFR mutation detection kit. Previous reports have described the details of the detection procedure [36].

### Statistical analysis

Continuous covariates such as metabolic parameters were compared against EGFR mutation status through the Wilcoxon rank-sum test. Differences in categorical variables including clinical parameters and PET/CT metabolic parameters among different mutation statuses were analyzed using Fisher's exact or chi-squared tests. A ROC curve was applied to obtain cutoff values for continuous variables to predict mutation status. Multivariate logistic regression analysis was used to analyze the independent predictors of EGFR+ vs. EGFR- status. Finally, the predictive value of the model based on the independent predictors was assessed by analyzing the area under the ROC curve (AUC). According to previous studies, an AUC value of at least 0.70 represents acceptable or good discrimination [37]. Two-sided p values <0.05 were considered statistically significant. All statistical analyses were performed using SPSS (version 20.0).
